# Adsorption of Hydrophobic Ions on Environmentally Relevant Sorbents

**DOI:** 10.3390/polym14153167

**Published:** 2022-08-03

**Authors:** Gergely Becskereki, George Horvai, Blanka Tóth

**Affiliations:** Department of Inorganic and Analytical Chemistry, Faculty of Chemical Engineering and Biotechnology, Budapest University of Technology and Economics, Műegyetem rkp. 3., H-1111 Budapest, Hungary; becskereki.gergely@vbk.bme.hu (G.B.); horvai.gyorgy@vbk.bme.hu (G.H.)

**Keywords:** adsorption, isotherm, environmental analysis, hydrophilic lipophilic balance, preconcentration, polar organic compound, POCIS

## Abstract

Environmental monitoring and remediation often requires the collection of harmful substances from aqueous solutions. Absorption with solids is a useful technique for binding such substances even at very low concentration levels. Many of these contaminants are weak acids or bases. Some novel, nonionic polymeric sorbents, such as hypercrosslinked polymers or polymers with balanced hydrophilic-lipophilic properties (HLB) have been found to bind weak acids and bases with high distribution coefficients even at pH values where these compounds are almost completely ionized (typically near pH 7). To understand this phenomenon and its practical consequences, we have experimentally studied the adsorption of ionizable weak acids and bases as a function of pH and ionic strength on a the OASIS^®^ HLB sorbent. Not surprisingly, the ionic forms of the weak acids and bases were found to be much less bound in the aqueous solution than their neutral forms. In spite of this, OASIS^®^ HLB binds weak acids and bases around pH 7 considerably better than typical hydrophobic sorbents. The high overall distribution coefficients around pH 7 could be explained by two factors. One is that on OASIS^®^ HLB, and on some other novel polymeric sorbents, the binding constant of the moderately hydrophobic neutral form is on the order of 100,000, i.e., much higher than on typical hydrophobic sorbents. Thus, even if the proportion of the neutral form in solution is only around 1% near pH 7, the adsorption of the neutral form is still significant. On the other hand, the binding of the apparently hydrophilic ionized forms occurs with distribution coefficients well above 100. The distribution coefficient of the ionic form appears to depend on ionic strength and the presence of competing ions. Adsorption of the ionic forms is found to be very similar to the adsorption of ionic surfactants. The pH dependence of the total adsorption of neutral and ionic forms together, is found to be steep around pH 7, and therefore the varying pH of natural waters may strongly influence the binding efficiency in practical applications, such as the collection (concentration) of contaminants or their passive sampling.

## 1. Introduction

Many organic compounds are easily adsorbed from water onto solid sorbents. This has made adsorption a preferential technique for collecting and concentrating organics from natural waters and all kinds of other aqueous solutions. For the treatment of large volumes of water, cheap inorganic sorbents are used. There are, however, many specialty applications in which polymeric sorbents offer distinctive advantages. For example, pesticide and pharmaceuticals contaminations of natural waters are concentrated on polymeric sorbents before instrumental analysis. In this case, the advantage of the polymers is that the contaminants from water are strongly bound, but easily released in a wash off step by a solvent, such as methanol. Inorganic sorbents are less suitable in this application because the release of the contaminants from them requires too harsh conditions, which may decompose the collected contaminants before the analysis could be made.

Environmental waters are often contaminated with organic compounds at low concentration levels, typically well below micromolar. Binding on a solid from such very dilute solutions requires very strong binding to the solid. Since the binding must be reversible, covalent bonds are avoided. Non-covalent binding may occur by van der Waals forces and by ionic forces. The latter are only effective with ion exchangers.

While polymeric ion exchangers have been in use for a long time, the use of neutral polymers for adsorption of ionized compounds was more limited until the 1990s. At this time two important innovative techniques became widespread. Hypercrosslinked polymers with uniquely strong binding capability for organics became commercially available under various trade names. Furthermore, porous polymers with mixed hydrophobic and hydrophilic groups (HLB, hydrophilic-lipophilic balance) began to be produced. Both of these novel types of polymers are based on the styrene-divinylbenzene (SDVB) structure, but modifications are introduced either by introducing extra crosslinks after the polymerization reaction (hypercrosslinking) or by copolymerization with a hydrophilic monomer (in the HLB type).

There are many reports in the literature in the last three decades about the practical applications of these novel polymers, but only a few studies went beyond the practical needs, and studied the general behavior of these polymers, particularly in the case of the HLB type. In an early study, Poole [1] used the Abraham model to discover the contributions of various binding forces to the adsorption of organics on OASIS^®^ HLB. Much more recently, Jeong et al. [2,3] studied the adsorption isotherms of many compounds on OASIS^®^ HLB.

An interesting feature of the novel polymers has been recognized, e.g., by Pichon et al. [4]. These authors could efficiently adsorb weak organic acids at neutral pH from natural waters. The authors [4] attributed their observation to the strong adsorption of the acid anions on the sorbent used. This was in apparent contrast to experience made with earlier porous SDVB sorbents and with reversed phase chromatographic sorbents, which would not bind these ions effectively at neutral pH.

The goal of the present work has been to investigate this phenomenon more closely. We have studied the binding of a weak acid and a weak base on a HLB type polymer, with special emphasis on the binding of the neutral and ionized forms. Our experiments show how solute concentration, medium pH, and ionic strength influence the binding of the neutral and ionized species, respectively. We show that in the vicinity of pH 7 the adsorption of both the neutral and the ionized form is significant, and their sum varies unexpectedly strongly in this pH range. This makes the efficiency of extraction more pH dependent than in case if only the ionic form were distributing. We also find that the distribution ratio of the ionic form depends on ionic strength and specific ion effects. We pay particular attention to the consequences of our results for applications such as preconcentration and passive sampling [5] of contaminants from environmental waters.

## 2. Theory

To support the subsequent discussion, we recall how the pH dependence of the adsorption of weak acids and weak bases on neutral sorbents is usually treated [6]. We also derive (in the Appendix A, Appendix B, Appendix C and Appendix D) useful relationships between the adsorptive distribution coefficient and the main parameters of two applications: (pre)concentration of organics and passive sampling of organics, respectively.

### 2.1. Phase Distribution of Weak Acids and Weak Bases

In a simple phase distribution system, a weak acid (or a weak base) is extracted from an aqueous solution into a hydrophobic medium. The weak acid or base may dissociate according to:

HA = H + A

In the case of an acid HA, H is the proton, and A the anion of the acid. In the case of a weak base A, H is again the proton, and HA is the protonated form of the base. (The charges of the species are not shown here.)

The usual assumptions for the distribution of a weak acid are:

1. The acid is monobasic.
2. The neutral form is soluble in both phases.
3. The ionic form is soluble only in the aqueous phase.
4. Neither the proton nor the hydroxyl ion can enter the hydrophobic phase.
5. There are no additional equilibria involved (e.g., the acid does not dimerize in either phase, and there are no ion exchange sites in the organic phase).

With these assumptions, two equilibrium equations may be written for a weak acid:

(1) $$K_{a}=\frac{\left[H\right]\left[A\right]}{\left[HA\right]}$$

and

(2) $$K_{HA}=\frac{{\left[HA\right]}_{org}}{{\left[HA\right]}_{aq}}$$

Here *K_a_* is the acid dissociation constant in the aqueous phase and *K_HA_* is the distribution constant of the neutral form of the acid, HA, between the two phases.

For a weak base, the dissociation equilibrium is described with the same equation as for the acid (Equation (1)); only HA is, in this case, the protonated form of the neutral base. The distribution equation is different from the acid case because here, the neutral basic form A distributes between the two phases:

(3) $$K_{A}=\frac{{\left[A\right]}_{org}}{{\left[A\right]}_{aq}}$$

If, in contrast to one of the above assumptions, the ionic form of the weak acid or base can also distribute between the two phases, then one also needs an equation describing the phase distribution of the ionic form. For example, in the case of a weak acid, the ionic form is the anion A. It is often assumed that the distribution of the ion A can be described with Equation (3), similar to the distribution of a neutral species. This assumption is not valid for liquid–liquid distribution, because ions with one charge sign cannot be extracted alone, i.e., without an equivalent amount of ions with the opposite charge. For adsorption with solids, however, electroneutrality may be maintained by ions of opposite charge remaining in the aqueous phase, but accumulating near the interface. This leads to a complex model, which is not further elaborated here. Instead, we derive here the formula found in the literature, by assuming that ions also have distribution *constants*.

When both the neutral and the ionic form can distribute between the two phases, the overall distribution coefficient, *D*, is as follows:

(4) $$D=\frac{{\left[HA\right]}_{org}+{\left[A\right]}_{org}}{{\left[HA\right]}_{aq}+{\left[A\right]}_{aq}}$$

With the help of Equations (1)–(3), *D* may be expressed as:

(5) $$D=\frac{K_{HA}{\left[HA\right]}_{aq}+K_{A}{\left[A\right]}_{aq}}{{\left[HA\right]}_{aq}+{\left[A\right]}_{aq}}=\frac{\frac{K_{HA}}{K_{a}}{\left[A\right]}_{aq}\left[H\right]+K_{A}{\left[A\right]}_{aq}}{\frac{1}{K_{a}}{\left[A\right]}_{aq}\left[H\right]+{\left[A\right]}_{aq}}=\frac{\frac{K_{HA}}{K_{a}}\left[H\right]+K_{A}}{\frac{1}{K_{a}}\left[H\right]+1}$$

**Figure 1:**
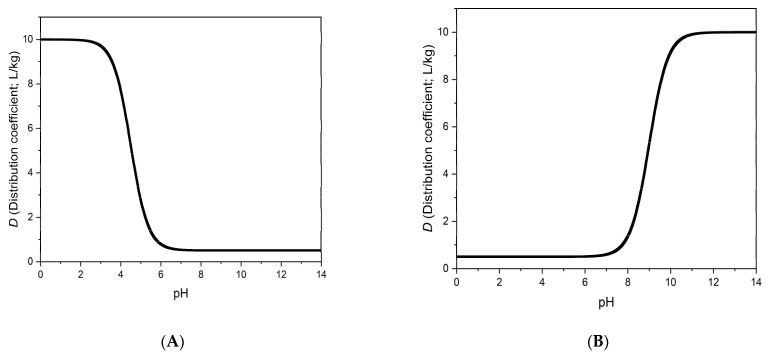
(**A**) shows the pH dependence of *D*, based on Equation (5) and assuming that *K_a_* = 1 ×10^−4.5^, *K_HA_* = 10, and *K_A_* = 0.5. The figure shows that at pH values much lower than p*K_a_* (which is 4.5 in the present case), the distribution coefficient is *D* = *K_HA_*, while at pH values well above the *pK_a_*, it is *D* = *K_A_*. The parameters chosen for (**A**) might represent a weak acid. With parameters that might represent a weak base (*pK_a_* = 9.0, *K_HA_* = 0.5, and *K_A_* = 10), one obtains the curve of (**B**). In either case, if the ionic form were not extracted, then its *K* value would be zero, and the curve would also go down to zero in the corresponding pH range.

The equation derived for *D* (Equation (5)) is widely used (eventually with minor corrections) in reversed phase liquid chromatography [6]. Figure 1 shows *D* as a function of pH for a weak acid (Figure 1A) and a weak base (Figure 1B), respectively. The parameters used would be typical for reversed phase liquid chromatography. Note that with the novel sorbents studied in the present work, the *K_HA_* and *K_A_* will be found to be orders of magnitude higher than in liquid chromatography, and the *K_HA_* to *K_A_* ratio will also be approximately one order of magnitude higher than in Figure 1. These quantitative differences will lead to important qualitative consequences.

Note that *D* is a distribution *coefficient*, while *K_HA_* and *K_A_* are distribution *constants*. The *K*-s are thermodynamic constants, while *D* depends on the experimental conditions, such as the pH.

An interesting feature of Equation (5) is that *D* is independent of the concentrations in either phase. One should also note, that according to Equation (5), the overall distribution coefficient *D* is the sum of two parts:

(6) $$D=\frac{\frac{K_{HA}}{K_{a}}\left[H\right]}{\frac{1}{K_{a}}\left[H\right]+1}+\frac{K_{A}}{\frac{1}{K_{a}}\left[H\right]+1}=D_{HA}+D_{A}$$

The first part shows the distribution coefficient if *K_A_* is zero, i.e., if the ion cannot distribute to the organic phase. In this case only HA distributes, so that *D* = *D_HA_*. (Note that *D_HA_* is different from *K_HA_*.) The second part (which might be denoted by *D_A_*) is an approximation for the distribution coefficient of the anion at pH values, which are high enough to make the first part negligible compared to the second. The condition for this approximation to be valid is:

(7) $$\frac{K_{HA}}{K_{a}}\left[H\right]<<K_{A}$$

### 2.2. Binding Capacity of Preconcentration Columns

The novel sorbents, which are the subject of this paper, have been often used to (pre) concentrate pollutants from natural waters. The preconcentration of water samples usually occurs on small packed columns, also called cartridges.

In Appendix A, a formula has been derived for estimating the maximum sample volume that can be percolated through such a column without analyte loss. Although the formula has been derived before, its derivation and the accompanying explanations are helpful for understanding the practical significance of our experimental results.

### 2.3. Maximal Sampling Time of Diffusive Sample Collection Devices

Another important application of the novel sorbents is in passive sampling of polar organic contaminants from waters. The passive samplers are exposed to the natural water for days or weeks, and then the collected amount of each contaminant is determined in the lab. The goal is to measure the time integral of the contaminant concentration during the exposure time. If the exposure time is too long, this goal cannot be achieved. In Appendix B, we derive a formula for the estimation of the maximum exposure time as determined by the contaminant’s distribution coefficient. This formula allows us to point out the difficulties of passive sampling at near neutral pH from natural waters.

## 3. Materials and Methods

### 3.1. Materials

OASIS**^®^** HLB, a hydrophilic-lipophilic balance sorbent, was purchased from WATERS (Waters™ Corporation, Milford, MA, USA) in Oasis^®^ HLB 6 cc Vac Cartridge form (200 mg Sorbent per Cartridge, 30 µm). Discovery^®^ DSC-18 (1 mL) SPE cartridge (Supelco™ Bellefonte, PA, USA) was obtained from Supelco (Distributed by Sigma-Aldrich, St. Louis, MO, USA).

Chemicals were mostly reagents of analytical grade. Details can be found in Appendix C.

### 3.2. Equipment

Laboratory consumables were obtained from Eppendorf^®^ (Eppendorf SE, Hamburg, Germany). Various volumes of Safe-Lock Eppendorf tubes, conical tubes (inert, Eppendorf Quality™, Eppendorf SE, Hamburg, Germany), and Eppendorf automatic pipettes were used in the experiments.

For sample preparation and other experiments, an Eppendorf^®^ Centrifuge 5430R (Eppendorf SE, Hamburg, Germany) and a Grant-bio^®^ PTR-35 (Grant Instruments (Cambridge) Ltd., Shepreth, Cambridgeshire, UK) multi-rotator were used under thermostatic conditions. For pH adjustments, a Thermo Scientific Orion^®^ 290A (Thermo Fisher Scientific, Waltham, MA, USA) portable pH/ISE/mV/T meter was used.

For the determination of the binding values and distribution coefficients of the substances under static or breakthrough conditions, HPLC measurements were carried out. We used an AGILENT 1100 series HPLC with Chemstation data acquisition software, a G1314A VWD detector, a G1316A column thermostat, and a G1330B autosampler thermostat, a G1328A autosampler, a G1312A binary pump, and a G1375A degasser (Agilent Technologies, Inc., Santa Clara, CA, USA). A Symmetry C18, 3.5 µm particle size 3.0 × 50 mm (Waters™ Corporation, Milford, MA, USA) column was used in HPLC separations.

A Precisa™ EP-225SM-DR (Precisa Gravimetrics AG, Dietikon, Switzerland) semi-micro analytical balance was used for weighing.

### 3.3. Sample Preparation

Adsorbent materials were collected from the commercial SPE cartridges. The SPE cartridge was pre-conditioned with methanol or methanol–water. The cartridge was washed with 3 × 1 mL MeOH/100 mg adsorbent, and after that, the cartridge was carefully cut or opened with a scalpel. The filter was removed from the cartridge, and the semi-wetted adsorbent was transferred to an inert Eppendorf tube or glass plate with a spatula. The sorbent material was then dried at room temperature in the ambient or in a desiccator.

### 3.4. Static (Batch) Adsorption and Breakthrough Measurements

Solid adsorbents were measured via analytical microbalance into Eppendorf safe lock tubes. Measured adsorbent weights were between 1.99 and 2.01 mg. Standards of substances were precisely weighed and diluted with methanol or buffer for stock solutions. After that, standard solutions were diluted to the chosen concentration (e.g., ketoprofen *c_0_* = 2 × 10^−5^ M) and simultaneously adjusted to the required composition (e.g., buffer concentration, buffer pH, organic–aqueous composition). When required, the ionic strength of the solutions was adjusted with KNO_3_ or Na_2_SO_4_.

The weighed adsorbent material was mixed with the corresponding concentration and composition solution in Eppendorf safe lock tubes during batch adsorption. The samples were shortly vortexed and then placed on a tumbling mixer, where they were kept under controlled room temperature (25.0 ± 1.0 °C.). After 2 h, the equilibrium state was reached (the change of *D* between 2 and 4 h was 1.8% (*n* = 6) for ketoprofen at pH 8.0). After this, the samples were centrifuged at 25 °C at 15,500 rpm for 15 min in a thermostated centrifuge. Supernatant from each sample was transferred into HPLC vials. Substance concentrations in the supernatant were measured by HPLC. The reproducibility of batch adsorption measurements was checked by six parallel measurements at one data point. The isotherm data points are averages of four measurements (two polymer samples with two HPLC measurements each).

Breakthrough was measured on 200 mg OASIS^®^ HLB SPE (Waters™ Corporation, Milford, MA, USA) cartridge. According to the manufacturer’s instructions, the column was washed with 5 mL methanol followed by 5 mL water. A total of 300 mL of the sample to be tested (5 × 10^−5^ M 2,4-dichlorophenoxyacetic acid in pH 7.0 phosphate buffer, 50 mM) was percolated through the cartridge, and collected in 5 mL fractions. Each fraction was analyzed separately by HPLC. The vacuum during the measurement was 5 mm Hg.

### 3.5. Chromatography

In the batch extraction measurements, the equilibrium solution concentrations were measured by HPLC. The HPLC methods used for the different compounds are described in Appendix D.

## 4. Results

The weak acid drug ketoprofen, the weak basic drug propranolol, and a neutral, drug-like hydrophobic substance, 1-(2-naphthyl)ethanol, were adsorbed on two different sorbents at several pH values and buffer concentrations from solutions made in water or in 20 *v*/*v*% methanol-water solution. The reproducibility of the adsorption measurements was checked with ketoprofen at pH 8.0, and was found to be 3.1% (*n* = 6).

### 4.1. Binding Isotherms at Different pH Values

Adsorption equilibria are primarily characterized by the binding isotherms. These show the adsorbed concentration, *q_eq_*, as a function of solution concentration, *c_eq_*, at equilibrium. For the weak acids and bases discussed here, *q* and *c* mean total concentrations, i.e., the sum of the concentrations of the neutral and the ionized species, respectively, in the given phase. Here we have chosen to plot log *q_eq_* against log *c_eq_* to be able to cover several orders of magnitude in concentration. In these log–log plots, most isotherms appeared to be nearly linear. This phenomenon will be discussed later.

**Figure 2:**
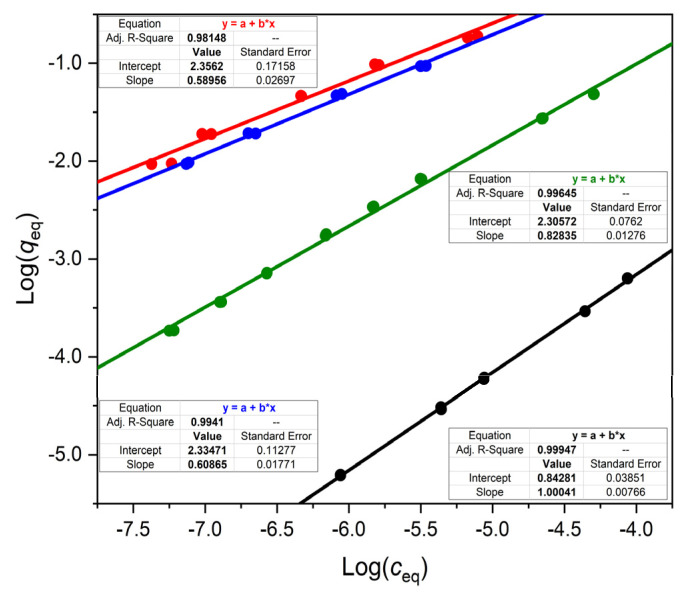
Comparison of ketoprofen isotherms under different adsorption conditions. All isotherms were measured in 20% methanolic buffer. The isotherms on OASIS^®^ HLB were measured at pH 3.2 (red), pH 4.3 (blue), and pH 7.0 (green), respectively. The ketoprofen isotherm on DSC-18 (a conventional C18 sorbent) was measured at pH 7.0 (**black**). Linear equations are shown in the figure with the corresponding colors.

**Figure 3:**
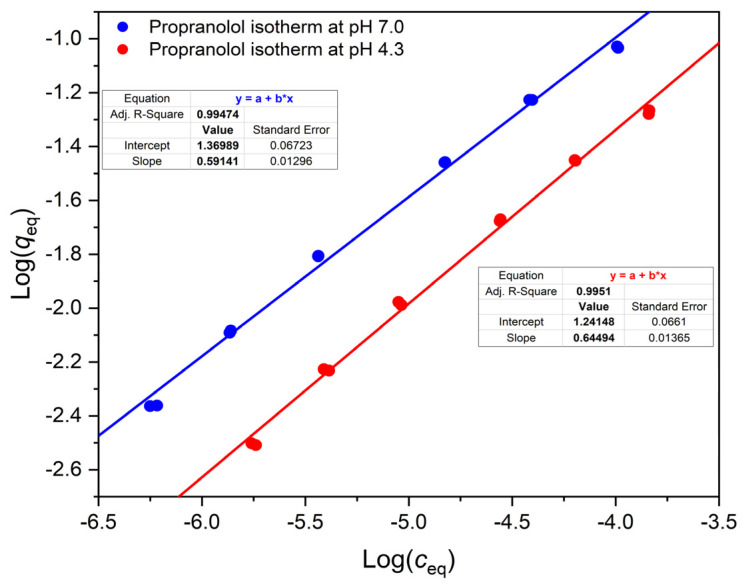
Comparison of propranolol isotherms under different adsorption conditions. The isotherms were measured in 20% methanolic buffers on OASIS^®^ HLB. at pH 7.0 (blue) and at pH 4.3 (red), respectively. Linear equations are shown in the figure with the corresponding colors.

**Figure 4:**
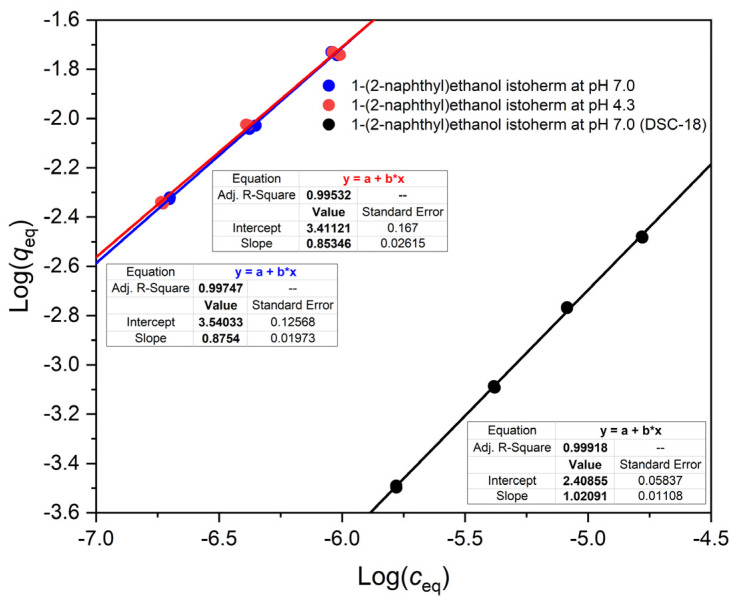
Comparison of 1-(2-naphthyl)ethanol isotherms under different adsorption conditions. All isotherms were measured in 20% methanolic buffers. Isotherms were measured on OASIS^®^ HLB at pH 7.0 (blue) and at pH 4.3 (red), respectively. The isotherm on DSC-18 (a conventional C18 sorbent) was measured at pH 7.0 (**black**). Linear equations are shown in the figure with the corresponding colors.

Since we studied a weak acid (ketoprofen), a weak base (propranolol), and—for comparison—a neutral compound (1-(2-naphthyl)ethanol), it was expected that the isotherms of the two ionizable compounds will be pH dependent, while that of the neutral compound will not depend on the pH. Figure 2, Figure 3 and Figure 4 show that this was indeed the case with the OASIS^®^ HLB sorbent. The isotherms of ketoprofen, a weak acid, shifted down at higher pH values (Figure 2), so that for a given *c* value *q* was less at higher pH. The binding of propranolol, a weak base, increased with higher pH (Figure 3), and the binding isotherm of 1-(2-naphthyl)ethanol, the neutral compound was practically independent from the pH (Figure 4).

The shapes of the individual isotherm lines in Figure 2, Figure 3 and Figure 4 appear to obey the Freundlich equation, i.e., the log–log plots are approximately linear. The slopes of the fitted lines are all less than one for the OASIS^®^ HLB material. In contrast to this, the slopes of the isotherms of both ketoprofen and the neutral compound 1-(2-naphthyl)ethanol, are exactly 1 on a typical reversed phase, C18, HPLC packing material, DSC-18 (Figure 2 and Figure 4).

Figure 2 and Figure 4 show also that the adsorption of either ketoprofen or 1-(2-naphthyl)ethanol is orders of magnitude higher on the OASIS^®^ HLB than on Discovery^®^ DSC-18, as was mentioned earlier in Section 2.1.

In the case of propranolol, which we were able to measure in a very wide concentration range (1 × 10^−8.3^ to 1 × 10^−3.8^), the Freundlich equation cannot be fitted well through this wide range. The fit is reasonably good in Figure 3, between 1 × 10^−6.2^ and 1 × 10^−3.8^ M concentration. Similar good fit could be achieved between 1 × 10^−8.3^ and 1 × 10^−5.5^ M (data not shown), but the parameters of the fitted lines were somewhat different in this lower concentration range. Notably, the slope increased from ca. 0.6 to ca 0.75 at both pH values.

### 4.2. pH Dependence of the Adsorption of the Neutral and Ionic Forms

The pH dependence of the overall distribution coefficient, *D*, of ketoprofen and propranolol, respectively, has been studied in more detail with OASIS^®^ HLB, by equilibrating 2 × 10^−5^ M solutions, made up in different pH buffers (all 20:80 *V*:*V* methanol:water). The solution volume and the sorbent mass was the same in each experiment. The measured distribution coefficients at the different pH values are shown in Table 1.

Table 1 shows that between pH 3.2 and 10.8, respectively, the distribution coefficient *D* of ketoprofen decreases, and that of propranolol increases by more than two orders of magnitude, similarly to the isotherm measurements. Yet these measurements, despit having been made under exactly identical conditions, apart from the pH adjustment, have a drawback. As is clear from the isotherm equations shown in Figure 2 and Figure 3, the distribution coefficients, *D* = *q*/*c*, are concentration dependent. Indeed, these equations are of the general form log *q* = log I + m × log *c*, where log I is the intercept and m the slope of the fitted straight line. Consequently, *q* = I × *c*^m^, and *D* = I × *c*^m−1^ or *D* = (I × *q*^m−1^)^1/m^. In the experiments of Table 1, the *starting concentration* of the compounds was always the same, but the *equilibrium concentrations*, *c* and *q*, varied largely between the experiments, due to the pH dependence of *D*.

Taking together the evidence from the isotherms and the pH dependence measurements, it can be seen that *D* depends both on the equilibrium concentration (in the solution or on the solid), and on the pH of the solution. Thus, if we wish to separate the pH dependence of *D* from its concentration dependence, we need to compare *D* values measured at different pH levels but at identical c or q values. Indeed, comparison at identical *q* values appears to be more logical, because the concentration dependence of *D* must be related to “filling up” the binding sites of the solid. Table 2 shows therefore the distribution coefficient *D* of ketoprofen at different pH values, but all at the same *q* value.

### 4.3. Buffer Concentration Dependence of the Adsorption of the Selected Drugs

**Figure 5:**
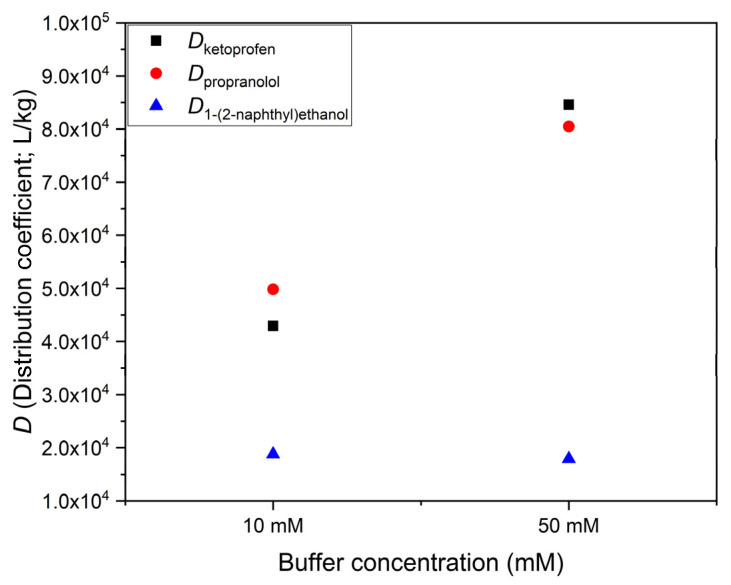
Buffer concentration effect on the distribution coefficients of ketoprofen, propranolol, and 1-(2-naphthyl)ethanol. The 1-(2-naphthyl)ethanol was measured in a 20% methanolic buffer at pH 7.0 (blue). Propranolol was measured in an aqueous buffer at pH 4.3 (red). Ketoprofen was measured in an aqueous buffer at pH 8.0 (**black**).

Figure 5 shows for all three compounds the dependence of *D* from the buffer concentration. Extraction experiments were made here with 2 × 10^−5^ M starting concentrations and with volume to mass ratio of 1000 µL/mg. The adsorption of the neutral compound does not depend on the buffer concentration. In contrast to this, the *D* values of ketoprofen and propranolol, respectively, are approximately two times higher in the 50 mM aqueous buffer than in the 10 mM buffer. (The isotherms described above had all been measured at 50 mM buffer concentrations, in 20 *v*/*v*% methanol–water medium.)

The effects are very similar with both ionizable compounds when measured at pH values where they are mainly in their respective ionic forms. However, since these ions are chemically very different and they have different charge signs, this suggests that the effect of buffer concentration is an ionic strength effect.

**Figure 6:**
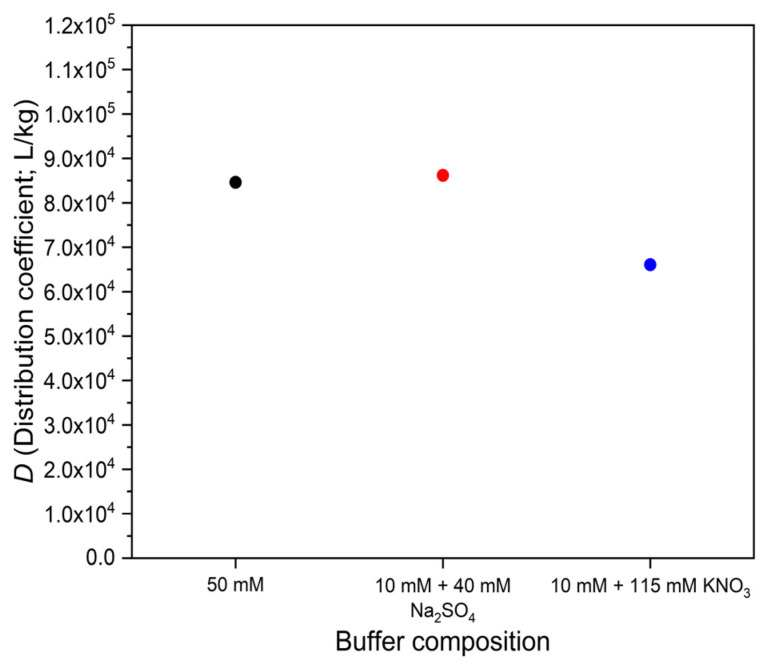
Ionic strength effect on the distribution coefficient of ketoprofen adsorption on OASIS^®^ HLB sorbent, from three phosphate buffer solutions of pH 8.0 and approximately equal ionic strength.

To test whether our experimental observations are indeed due to ionic strength differences, we have compared the adsorption of ketoprofen from the 10 mM phosphate buffer of pH 8.0 with adsorption from an electrolyte consisting of the same 10 mM phosphate buffer plus 40 mM Na_2_SO_4_. The ionic strength of the latter mixture is about the same as that of the 50 mM phosphate buffer. We have found that the effect of the mixture with Na_2_SO_4_ is about the same as that of the 50 mM phosphate buffer, i.e., the binding increases about twofold, compared to the 10 mM phosphate buffer (Figure 6, compared with Figure 5).

However, when KNO_3_ was added to the 10 mM phosphate buffer in a concentration of 115 mM, which had adjusted the ionic strength to that of the previously tested 50 mM phosphate buffer, we obtained a different result. The observed effect on the adsorption of ketoprofen was still positive but much less than with the 50 mM phosphate buffer or with the 10 mM buffer + 40 mM Na_2_SO_4_ mixture (Figure 6).

### 4.4. Breakthrough Curve

The breakthrough curve of 2,4-dichlorophenoxyacetic acid, a pesticide representing environmental hazards, has been measured on an OASIS^®^ HLB 200 mg cartridge from a pH 7.0 buffer. The *D* value calculated from the breakthrough curve (1200) agreed closely with the *D* measured in the static adsorption (isotherm) measurement (1120). This indicates that our results, obtained from static adsorption data, may be applied to solid phase extraction on the OASIS^®^ HLB cartridges.

## 5. Discussion

In the last decades, several new polymeric sorbents have been invented, which can bind strongly to almost any weakly hydrophobic compound. Two important groups of such polymers are the hypercrosslinked polymers [7] and the hydrophilic–lipophilic balanced (HLB) polymers [1]. These polymers share the strong binding property with activated carbon and other carbon materials, but in contrast, binding on the novel polymers is more reversible, and the bound substances may be eluted under mild conditions. This makes the novel polymer sorbents suitable for many practical applications. These include analytical sample preconcentration, even at extremely low analyte concentrations, and the passive sampling of environmental and other waters. With more recent and very expensive equipment, one may measure concentrations in this very low range without preconcentration [8], but this does not make preconcentration generally superfluous, not even with MS detection [9]. There are also many other possible applications beyond analytical chemistry. Some of these, including the preparation of nanocomposites, have been reported by the Davankov group [10]. The possibilities may be further extended by the invention of a simple, versatile, fast, and green synthesis method involving hypercrosslinked polymers [11].

It is interesting to note that HLB polymeric sorbents were developed in the first place to make them directly water-wettable, without prewetting with an organic solvent [Waters™ Corporation, Milford, MA]. The OASIS^®^ HLB sorbent, analyzed in the present work, is not a hypercrosslinked polymer, yet it is also highly adsorptive, well beyond the level that might be expected merely on the basis of its 800 sqm/g specific surface area. It is even more surprising that this styrene divinylbenzene (SDVB) copolymer, which contains a large proportion of hydrophilic N-vinylpyrrolidone groups, has similar binding properties to the purely SDVB hypercrosslinked sorbents.

### 5.1. Binding Isotherms

Before any practical application of a novel sorbent, it is useful to measure its adsorption isotherm. In case of weak acid or weak base adsorbates, the isotherms should be measured at different pH values.

The isotherms of modern liquid chromatography packing materials are usually linear over many concentration decades. It seems that the hypercrosslinked and HLB sorbents have been used very often without checking their isotherms, and therefore assuming isotherm linearity. Our experiments show that this assumption is not generally warranted. Overlooking isotherm nonlinearity may lead to serious analytical and technological errors.

We have found that the isotherms of a weak acid, a weak base and a neutral substance are all nonlinear on OASIS^®^ HLB. This is reflected by the slope *m* of their Freundlich lines (in log *q*-log *c* plots), which was less than 1.

If the slope in the log–log plot is significantly different from 1, then the *q* vs. *c* isotherm is a power function, and thus it is nonlinear. This has many consequences [12]. If *m* < 1, then the distribution coefficient of the substance is decreasing as the concentration of the substance increases. As a consequence of this, the shape of the breakthrough front in solid phase extraction will be sharper (more sudden) than with a linear isotherm. Moreover, if the analyte concentration is higher, the breakthrough will occur sooner. All this needs to be considered when optimizing an SPE method.

Jeong et al. [2,3] and Huysman et al. [13] have also noted that the Freundlich exponents of many compounds deviated significantly from 1 on OASIS^®^ HLB. While we have only observed slope (*m*) values less than 1 with OASIS^®^ HLB, these other investigators reported *m* values both below and above 1. It is interesting that these authors observed *m* > 1 (in their notation *n* < 1, where *n* = 1/*m*) values. Isotherms with *m* > 1 are usually a sign of multilayer adsorption [12], but this appears to be unlikely at the very low *c* values in the cited works. In any case, if the Freundlich exponent deviates from 1, the distribution coefficient varies with the analyte concentration, and this has important practical consequences, as pointed out above.

In the present work, we have particularly sought to study adsorption up to higher *q* values than others before us, i.e., even well above 0.01 mol/kg adsorbed concentration. Thus, we could show that OASIS^®^ HLB also has unique properties (very strong adsorption, even of ions, and nonlinear isotherm) in this range. This is a concentration range where novel technical applications can be imagined, including interesting chemical reactions with adsorbed substances, on a preparative scale.

### 5.2. pH Dependence of Adsorption

An important feature of the novel sorbents which have been studied in this paper is their capability to adsorb ionizable organic compounds, such as many drugs and pesticides, at pH values where their ionic form dominates in solution, e.g., 2 pH units above their *pK_a_* values in case of weak acids. This property allows the simultaneous collection of neutral, weakly acidic and weakly basic compounds at near-neutral pH values. This is very important because multianalyte analyses, typically with chromatography, are in great demand these days.

In Section 4.1 and Section 5.1, it has been shown that the isotherms of a weak acid and a weak base are highly pH dependent on OASIS^®^ HLB. In the case of the acid, ketoprofen, binding at pH 7.0 was in the whole studied concentration range about two orders of magnitude less than at pH 3.2. Yet, the binding at pH 7.0 was still substantial, with the distribution coefficient around 1000. Since in the aqueous phase more than 99% of ketoprofen is in ionic form at pH 7.0 (see Table 2), one might think that at this pH (and already from about pH 6) it would be almost exclusively the ionic form which is adsorbed. This view was indeed adopted when the novel adsorbents were first used for binding weak acids at neutral pH [4].

We thought, however, that one cannot decide simply from the relative proportions of the ionic and the neutral forms (in the aqueous phase) the relative contributions of the ionic and neutral forms to the observed overall distribution coefficient. Indeed, according to Equation (6), the relative contributions of the two species to *D* are:

(8) $$\frac{D_{HA}}{D_{A}}=\frac{\frac{K_{HA}}{K_{a}}\left[H\right]}{K_{A}}=\frac{K_{HA}}{K_{A}}\frac{\left[H\right]}{K_{a}}$$

As Equation (8) shows, at a given pH, the relative contributions of the neutral and the ionic forms to *D* depend on both the pH–*pK_a_* difference (which is equal to −log ([*H*]/*K_a_*)), and on the ratio of the distribution constants *K_HA_* and *K_A_*. By way of an example: if *K_HA_*/*K_A_* = 200 (the neutral form is much better adsorbed than the ion), and if [*H*]/*K_a_* = 0.01 (i.e., pH = *pK_a_* + 2), then the neutral to ionic ratio, [*HA*]/[*A*] is 0.01 (by virtue of Equation (1)), but *D_HA_*/*D_A_* is 2. This means that the neutral form, which accounts for only 1% of the compound in solution, has a two times higher contribution to *D* than the ionic form, which accounts for 99% in the solution. In other words, the concentration ratio of the neutral to the ionic form is 1:99 in the solution, but 2:1 in the adsorbed state.

Equation (6) also shows that even if the ionic form had zero distribution constant, the overall distribution coefficient might be high at pH values, where the ionic form is dominant in the solution. Indeed, if *K_A_* = 0, Equation (6) gives:

(9) $$D=\frac{\frac{K_{HA}}{K_{a}}\left[H\right]}{\frac{1}{K_{a}}\left[H\right]+1}$$

If, for example, pH = *pK_a_* + 2, so that [*H*]/*K_a_* = 0.01, then the denominator in Equation (9) is 1.01≈1, and *D* = 0.01 × *K_HA_*. On the novel sorbents, *K_HA_* values are very high, on the order of 1 × 10^5^–1 × 10^6^. Thus, even if the nonadsorbing ionic form dominates in the solution, *D* may be on the order of 1000–10,000, which are very high values.

Our experimental results obtained with the weak acid ketoprofen, and presented in Table 2, confirm these conclusions. To prove this, we needed to calculate the values of *K_HA_*, *K_A_* and *pK_a_* from the experimental data in Table 2. Before showing how this was done, we shall point out the unique properties of OASIS^®^ HLB which follow from Table 2.

The data in Table 2 are indeed remarkable. At pH 3.2, *D* is almost 200,000. This is a very high value. In HPLC a typical *D* value would be only around 10. In solid phase extraction, the high *D* value of ca. 200,000 would indicate a breakthrough volume of 200 L on a sorbent packing of only 1 g mass (according to the formula derived in Appendix A). However, even at pH 7, i.e., for “neutral” water samples, *D* of ketoprofen has been 974, which allows the percolation of 974 mL of sample through a 1 g extraction packing without full breakthrough. Thus our data support the observations made by others [4] that sufficiently “hydrophobic” weak acids may be extracted with the novel sorbents even from neutral solutions with high recovery (little breakthrough).

The values of *K_HA_*, *K_A_* and *pK_a_* may be calculated from the data in Table 2 in the following way. Since at pH 10.0, *D* is approximately equal to *K_A_*, as follows from Equation (6) for a weak acid with pKa well below 7. Thus, *K_A_* is about 468. One can also see from Equation (6), that at pH 3.2 and 4.3, *D_A_* is less than *K_A_* = 468, and thus negligible compared to the *D* values measured at pH 3.2 and 4.3, respectively, which are both above 100,000. Thus, at pH 3.2 and 4.3 *D* is approximately equal to *D_HA_*. This allows us to calculate *K_HA_* and *K_a_* from the *D* values measured at pH 3.2 and 4.3, respectively. The values obtained were *K_HA_* = 211,173 and *K_a_* = 5.17 × 10^−5^ (*pK_a_* = 4.29). Having these two constants, one can calculate from the first part of Equation (5) (which is formally the same as Equation (6)) the pH dependence of the distribution coefficient *D_HA_* for the neutral form of ketoprofen. The respective values, calculated for pH 6.0, 7.0 and 10.0 are shown in Table 2. We see, that at pH 7.0 the calculated *D_HA_* is still substantial, its value being 407, which is not negligible compared to the measured value, 974.

An estimation of the distribution constant of ketoprofen’s ionized form, *K_A_*, from Table 2 is difficult, but an approximate value can be obtained. This can be achieved if we compare the data in Table 2 for pH 7.0 and 10.0. According to Equation (6), the difference between the measured *D* and the calculated *D_HA_* values at any pH should show the contribution of the anions to the distribution coefficient. This contribution should be almost constant in this pH range, because here *[H]*/*K_a_* << 1, so that *D_A_*≈*K_A_* = constant. However, one can see that the apparent ionic contribution decreases substantially with increasing pH. If we assume that at pH 10.0 we are really measuring *K_A_*, the distribution constant of the anion, then *K_A_* is only 468, but it is certainly not higher than about 1000.

In any case, the estimated contribution of the neutral form of ketoprofen, *D_HA_*, to the overall distribution coefficient, *D*, is not negligible even at pH 7.0, and at pH 6.0 it is even higher, essentially equal to the overall *D* of 3930 (Table 2). Thus, at pH 6.0 and 7.0 the contribution of the neutral form to *D* is substantial, and one cannot speak of pure ion-extraction. This experimental result is well explained by our theoretical calculations. The ratio of the experimental *K_HA_* to *K_A_* (211,173 to 468, i.e., 451) is indeed very high. Thus, on OASIS^®^ HLB the distribution constant of the neutral form of ketoprofen is 451 times higher than the distribution constant of the ketoprofen anion, and therefore even at pH values above *pK_a_* + 2, the overall distribution is far from purely ionic. This situation may apply to many other environmentally relevant weak acids, which are fairly hydrophobic in their neutral form. For hydrophobic bases, an analogous conclusion is also warranted, as seen in Table 1 for the case of propranolol (*pK_a_* being about 9.5): the contribution of the neutral (basic) form to *D* must be significant even if the pH is more than 2 units below the *pK_a_*, since in our experiment at pH 7.0, *D* is 4270, which is much higher than 1100, the *D* at pH 4.3.

An important practical consequence of our theoretical results and of the experimental data in Table 1 and Table 2 is that the distribution coefficient of a weak acid (or a weak base) may vary substantially within the “neutral” pH range (between ca. pH 5.5 to 8.5). The *D* values of ketoprofen at pH 6.0 and 7.0, which are ca. 4000 and 1000, respectively, show a fourfold change between pH 6.0 and 7.0. In real life applications, e.g., when working with natural water samples, which may have different pH values in this pH range, the breakthrough volumes may also vary by a factor of four (see Appendix A), and therefore recoveries may vary from sample to sample. This problem is not limited to ketoprofen, it is a general problem, as shown by the following example. Let us assume, that *K_HA_*/*K_A_* = 100, which appears to be a typical order of magnitude for the novel sorbents. The *pK_a_* of the acid should be about 5, which is a good approximation for many carboxylic acids. Then, according to Equation (5), *D* is equal to 10 × *K_A_* at pH 6.0, 1.98 × *K_A_* at pH 7.0 and 1.1 × *K_A_* at pH 8.0. This is a nearly tenfold change of *D* between pH 6.0 and 8.0.

### 5.3. Buffer Concentration Dependence of the Adsorption of Ions

Our experiments with different buffer concentrations (Figure 5) showed that the distribution coefficients of the ionizable compounds (ketoprofen and propranolol) were sensitive to buffer concentration at pH values, where the contribution of the neutral form was small compared to the ionic form. The neutral compound (1-(2-naphthyl)ethanol) used for comparison did not show such effect. This indicates that salting out is unlikely to be the cause of the buffer concentration effect. Ion exchange on some unsuspected ionic impurities of the polymer is also unlikely, because that would require that the adsorption decreases at higher buffer concentration. Changes in the activity coefficients of the adsorbable ions due to the increased ionic strength would also decrease the binding by reducing the activity of the adsorbable ions. The most likely remaining explanation is the electrical effect of the adsorbed ions, discussed, for example, by Hagglund and Stahlberg [14]. Adsorbed ions make the sorbent surface charged, and the required countercharges accumulate in the solution, near the interface. The capacitance of the resulting double layer increases with higher buffer concentration. This, in turn, allows more ions to be adsorbed, so that *D* can indeed increase. In this model, it is essentially the increased ionic strength of the more concentrated buffer which increases the capacitance and *D*. We tested this conclusion by adjusting the ionic strength of the more dilute buffer to that of the more concentrated buffer by using Na_2_SO_4_. The observed effect (identical increase in *D*) confirmed our expectation.

We have noted, however, that other authors [2,13] have made different observations from ours: the effect of ionic strength was small or even opposite to the one seen by us. Therefore, we have tested an ionic strength adjustment electrolyte different from the first-used Na_2_SO_4_. When adjusting the ionic strength of the more dilute buffer with the necessary amount of KNO_3_ (Figure 6), *D* had not reached the same value as with Na_2_SO_4_. We believe that the ionic strength effect was, in this case, the same as with Na_2_SO_4_, but the relatively hydrophobic nitrate ion had also a second effect: it competed with the ketoprofen anion for binding to the sorbent. Notwithstanding the exact reason, one may say, that ionic strength effects on the adsorption may be modified or even obscured by other, less known effects.

### 5.4. Mechanism of Adsorption

Our experimental observations show that binding of ionizable compounds, particularly of their ionic form, by the novel sorbents must be related to hydrophobicity, but there must be some other factors, too. Hydrophobicity refers to the change of standard chemical potential when a species is transferred from one bulk phase into another bulk phase. In contrast to this, an adsorbed molecule or ion is generally not (fully) immersed into the solid sorbent. Rather, it is attached to the sorbent surface but remains partly in contact with the solvent. As is known for surfactants, hydrophilic “headgroups” may remain fully solvated by water.

Adsorption of (hydrophobic) ions on a neutral (i.e., not ion exchanger type) solid sorbent is not easy to treat theoretically. Gritti [15] has recently summarized the different approaches for the quantitative prediction of retention, and thus of adsorption, in HPLC. He concluded that all known methods need improvement concerning the retention of ionizable solutes. One should add to this that the models for describing the adsorption of ionic surfactants at the water–air interface, which appears to be simpler than adsorption with solids, are still hotly debated [16], and new approaches are appearing [17]. For this reason, only a qualitative model will be used here. Nonetheless, the models used to describe surfactant adsorption on the water–air interface have relevance here, as the ionic form of many ionizable drugs have been shown to be reasonably good surfactants [18,19].

The ionized forms of the head groups of weak acids and bases are much more hydrophilic than in the neutral forms. This explains the more than one-hundredfold lower adsorption constant of the ionic form that was observed, compared to the neutral one. At near-neutral pH, both forms may comparably contribute to the overall adsorption: the ionic form because of its high relative abundance, while the neutral, much less abundant form by its much higher distribution constant.

The unique property of the novel (hypercrosslinked and HLB) sorbents, that they can strongly (with *D* between 500 and several thousand) bind weak acids and bases around pH 7, where their ionic forms dominate in solution, can also be explained by our results. The interaction of these novel polymers with the hydrophobic molecules, as expressed by their *distribution constants*, is 2–4 orders of magnitude stronger than that observed with comparable chromatographic sorbents, e.g., C18 modified silicas. Thus, the *distribution coefficient* of the neutral form is still quite high at around pH 7. On the other hand, due to the very strong adsorption of the hydrophobic part of the molecule, even the ionized form is significantly adsorbed (although its *distribution constant* is about two orders of magnitude lower than that of the neutral form). These effects are even more pronounced in purely aqueous solutions, than in the 20% methanolic solutions used in the present study. Approximate measurements showed that in the absence of methanol, the adsorption of the neutral form would have been unmeasurably high by UV-HPLC.

## 6. Conclusions

We studied the adsorption equilibria of a weak acid and a weak base on a novel, commercially available polymeric adsorbent with unique properties (OASIS^®^ HLB). Our goal has been to better understand the very strong, and in practical applications widely utilized, adsorption of ionizable organic compounds with OASIS^®^ HLB. In published reports directed towards practical applications, such as the preconcentration of contaminants from water, the recovery of the contaminants has often been studied, but recovery is a complex quantity. Our equilibrium studies can better serve as guidelines for method development with OASIS^®^ HLB and other sorbents, such as hypercrosslinked polymers.

The adsorption isotherms (i.e., the *q* vs. *c* functions) of the two ionizable compounds have been found to be nonlinear power functions in a wide concentration range, and at various pH values. The Freundlich isotherm equation was approximately valid, with slope *m* < 1. This is important in preconcentration applications, because breakthrough curves can be expected to be sharp. On the other hand, *m* < 1 predicts that recoveries of the less-retained compounds may be concentration dependent.

The distribution constants of the neutral and the ionic forms of ionizable compounds were found to differ by about two orders of magnitude. Notably, however, due to the very high distribution constant of the neutral form (above 100,000 in the presence of 20% methanol), even the ionic form has a sufficient distribution constant for (pre)concentration applications.

Nevertheless, near pH 7, where the ionic form is dominant in solution, the adsorption of the neutral form is also appreciable, due to its much higher distribution constant. The simultaneous adsorption of the two forms leads to a significant pH dependence of the overall distribution coefficient around pH 7. This may cause unexpected variations of system performance in practical applications, where pH may vary from sample to sample or with time (e.g., in natural waters). Another factor of uncertainty may be the observed dependence of the distribution of the ionic form on the main electrolytes in the solution.

The adsorption mechanism of both the neutral and ionic forms appears to be similar to the adsorption of surfactants. The hydrophobic part of the molecule interacts closely with the sorbent, while the hydrophilic head group remains (fully or partly) solvated by water. This model can easily explain the large difference between the distribution constants of the neutral and ionic form. It can also explain why the “hydrophilic” (water soluble) ionic form is strongly adsorbed from water.

## Figures and Tables

**Table 1:** Experimental overall distribution coefficients (*D*) of ketoprofen and of propranolol, in extraction experiments with 2 × 10^−5^ M starting concentration and with volume to mass ratio of 1000 µL/mg.

pH	Ketoprofen *D*	Propranolol *D*
3.2	185,100	-
4.3	95,600	1100
6.0	3931	-
7.0	-	4270
8.0	890	-
10.0	-	164,500
10.8	-	171,300

**Table 2:** Experimental overall distribution coefficients (*D*) of ketoprofen, determined from its pH dependent isotherms, at constant *q* (*q* = 0.0156 mol/kg, log *q* = −1.8077). The calculated distribution coefficients of the neutral (*D_HA_*) and ionic (*D_A_* = *D* − *D_HA_*) forms of ketoprofen, respectively, are also shown. (The small negative value of *D_A_* at pH 6.0 is contributed to uncertainties in the models used, and may be considered to be 0). The calculated pKa is 4.29.

pH	Ketoprofen *D*	Ketoprofen *D_HA_*	Ketoprofen *D_A_*	% HA in Solution
3.2	195,170	195,170	0	92.4
4.3	103,920	103,920	0	49.2
6.0	3930	(4005)	(−75)	1.9
7.0	974	407	566	0.2
10.0	468	0.41	468	0

## Data Availability

The data presented in this study are available on request from the corresponding author.

## Appendix A

### Binding Capacity of Preconcentration Columns

In practice, water samples are percolated through a pre-wetted sorbent column for preconcentration. During the percolation, the contaminants are adsorbed on the column. Unless the flow rate of the sample through the column is high, equilibrium adsorption will occur. This is assumed here to be the case. In the next step of the preconcentration, the contaminants are eluted from the column with a small volume of a strongly eluting solvent, typically methanol.

During the sample percolation process, the adsorption of the contaminants begins at the top of the column, and as more and more sample passes through the column, the sorbent “saturation” proceeds from top to bottom. During this time, the water leaving the column is essentially free from contaminants. If the sample volume is too large, the contaminants, beginning with the least strongly bound ones, can “break through”, i.e., the contaminants begin to appear in the water leaving the column. If even more water sample is applied to the column, the concentration of the contaminants increases in the effluent water until it reaches a plateau, which is the same concentration as that in the contaminated sample. At this point, the sorbent in the column is in equilibrium with the original sample concentration of the contaminant.

It is very important that upon full breakthrough of a contaminant, the concentration of the adsorbed contaminant on the sorbent in the column is uniform, and it is the concentration in equilibrium with the original sample concentration. There are two important consequences of this. First, that the column is not saturated in the sense that it would not be able to bind more of that contaminant. If a sample with a higher contaminant concentration is percolated until breakthrough, the column will bind more contaminant. This is so because the equilibrium relationship between the sample concentration and the adsorbed concentration, the adsorption isotherm, is a monotonously increasing function of the sample concentration. The second consequence is, that the breakthrough volume, i.e., the volume of the sample where the breakthrough occurs, depends on the contaminant’s chemical properties, and it may also depend (if the adsorption isotherm is not linear) on the contaminant concentration.

One may define the breakthrough volume in two different ways. One is the sample volume at which breakthrough begins, i.e., when the contaminant concentration in the column effluent is already significant, e.g., 1% of the sample concentration. This breakthrough volume has obvious practical importance. Yet it depends not only on the binding equilibrium but also on the dispersion in the column. An alternative definition of the breakthrough volume is the sample volume where the contaminant concentration in the column effluent is 50% of the sample concentration. This breakthrough volume has been found to depend only on the adsorption equilibrium, according to:

(A1) $$V_{br}=\frac{m_{s}q_{s}}{c}+V_{0}$$

where *V_br_* is the breakthrough volume, *V*_0_ is the dead volume of the column, *c* is the sample concentration, *m_s_* is the sorbent mass in the cartridge, and *q_s_* is the contaminant concentration on the sorbent. Since *q_s_/c = D* and *V*_0_ is typically much less than *V_br_*, we obtain the approximation:

(A2) $$V_{br}=Dm_{s}$$

This equation shows that for a given sorbent mass, the breakthrough volume is directly proportional with the distribution coefficient of the contaminant. In other words, compounds with high *D* do not easily break through, since they are strongly retained on the column. On the other hand, the lower is *D*, the sooner will breakthrough occur, unless the sorbent mass is increased. A further conclusion to be made from this equation is that the breakthrough volume is independent from the sample concentration unless *D* itself is concentration dependent. Our experimental data show that in some cases, *D* decreases at higher sample concentrations, so that the breakthrough volume also decreases with higher sample concentration.

The preconcentration procedures need to be optimized. The typical goal of the optimization is to prevent (or minimize) breakthrough for all important components, but without using too much sorbent material. These two, contradicting goals lead to the frequently noticed optimization strategy, that just enough sorbent is employed to prevent the breakthrough of the least bound contaminant. The danger of this approach is, that any unexpected variations in the sample properties may lead to a drop of *D*, and thus to breakthrough, even if no breakthrough had been observed during method validation. The results, presented in our paper, show that this can be a real problem.

## Appendix B

### Maximal Sampling Time of Diffusive Sample Collection Devices

An important environmental application of the novel sorbents, such as OASIS^®^ HLB, is in passive sampling of polar organic contaminants from environmental waters. A widely used such device is the POCIS (polar organic chemical integrative sampler) [5]. This simple device consists of a flat chamber, densely filled with a sorbent. In the interstitial space between the particles there is water. One side of the chamber consists of a membrane which allows the passage of molecules and ions. The chamber is submersed into a natural water, such as a river, for a predetermined time, e.g., for 28 days. During this time, the contaminants of water can diffuse into the chamber, where they are bound by the sorbent. Under certain conditions (see below), the collected amount of any adsorbable contaminant is proportional to the time integral of its concentration in the river water during the time of deployment.

We will show now, that the maximum sampling time until which the sampling may be considered integrative, depends on the distribution coefficient, *D*, of the particular contaminant. This is very important, because if *D* is less during actual deployment than during method development, then false measurement results will be produced.

In passive samplers, such as POCIS, the influx of contaminants into the sampling chamber occurs by diffusion. The hourly influx of a contaminant is approximately proportional to its concentration difference between the outer and inner solution. As long as the inner concentration, *c_int_*, does not reach a certain fraction (say 30%) of the external concentration, *c_ext_*, the influx is approximately proportional with the external concentration itself. Thus, during time, *t*, the amount of contaminant arriving into the chamber is proportional to the time integral of the external concentration during time *t*.

Due to the very high distribution coefficients of many polar organic contaminants on OASIS^®^ HLB, practically all of the contaminant which has diffused into the chamber during time *t*, is bound on the sorbent. Thus, the concentration, *c_int_ (t)*, of a contaminant in the internal solution in the chamber at time *t* can be calculated as:

(A3) $$c_{int}\left(t\right)=\frac{n\left(t\right)}{m_{sorb}D}$$

where *n(t)* is the amont of the contaminant in the chamber after time *t*, *m_sorb_* is the mass of sorbent and *D* is the distribution coefficient of the contaminant.

As noted above, the maximum time of deployment is limited by *c_int_*, which should not exceed a certain fraction of the external concentration. Since *c_int_* is inversely proportianl to *D*, this limit will be reached sooner if *D* is less than expected. For example, if the pH of the sampled water (and consequently of the internal solution) differs from the pH of the calibration solution, and *D* changes accordingly, then erroneous results may be obtained.

## Appendix C

### Appendix C.1. Chemicals

Water was supplied by a MILLI-Q^®^ Direct 8 Water Purification System provided by Merck. For HPLC and batch adsorption measurements, high purity acetonitrile and methanol (HPLC grade, LiChrosolv^®^ Reag. Ph Eur.) were used from Merck. The pH/ISE/mV/T meter was calibrated using buffer reference stock solutions of pH (4.00 ± 0.01), pH (7.00 ± 0.01), and pH (10.00 ± 0.01), supplied by Sigma-Aldrich (Sigma-Aldrich, Saint Louis, MO, USA). Ketoprofen drug standard (≥ 99.4%) and 2,4-dichlorophenoxyacetic acid (≥98%) were bought from Alfa Aesar^®^ (now branded by Thermo Fisher Scientific, Waltham, MA, USA). (S)-(-)-α-methyl-2-naphthalenemethanol (for short: 1-(2-naphthyl)ethanol) (98%) and (±)-propranolol hydrochloride (≥99%) were ordered from Sigma-Aldrich (Sigma-Aldrich, Saint Louis, MO, USA). For buffer solutions, formic acid (98–100%; LC-MS grade), ammonia solution (32%, EMPLURA^®^), ammonium-formate (≥99%), and ammonium-acetate (≥98%) were supplied by Sigma-Aldrich (Sigma-Aldrich, Saint Louis, MO, USA). Sodium dihydrogen-phosphate (≥99%); di-sodium hydrogen phosphate (≥99%); sodium carbonate anhydrous (≥99%); and sodium hydrogen carbonate (≥99%) were supplied by Fluka^®^ (Honeywell Research Chemicals; Honeywell International Inc., Charlotte, NC, USA). Sodium hydroxide (≥99%) was bought from Molar chemicals. Sodium sulfate (anhydrous (≥99%)) and potassium nitrate (≥99%) were provided by Sigma-Aldrich (Sigma-Aldrich, Saint Louis, MO, USA).

### Appendix C.2. Preparation of Buffers

For pH 7.0 and pH 8.0 measurements, phosphate salts were mixed in appropriate ratio and concentration, diluted with pure water. After that, the pH was adjusted with sodium hydroxide solution to the required pH with the help of the pH meter. For the pH 10.0 buffer, carbonate salts were used and adjusted similarly to phosphate buffers. For the low pH range, sodium-formate buffers were used. pH 3.2 and pH 4.3 buffer solutions were made by adjusting the pH of formic acid solutions of appropriate concentration to the required pH with sodium hydroxide.

## Appendix D

### Chromatographic Methods

Sample injection volumes were 10 µL. The autosampler temperature was 25 °C, and the column temperature was 26 °C.

For ketoprofen, propranolol, and 1-(2-naphthyl)ethanol measurements, the Symmetry C18; 3.5 µm; 3.0 × 50 mm column was used.

The other chromatographic conditions were as follows:

Ketoprofen HPLC: The isocratic eluent was 40:60:0.2 *V*:*V* acetonitrile: water: HCOOH (formic acid) mixture, with a 0.6 mL/min flow rate. The detection wavelength was 260 nm. The retention factor was *k*_ketoprofen_ = 5.15.

Propranolol HPLC: the isocratic eluent was 25:75 *V*:*V* acetonitrile: buffer (aqueous buffer containing 0.05 M ammonium-formate and 0.1 *V*/*V*% HCOOH, pH3, 95) with a 0.6 mL/min flow rate. The detection wavelength was 289 nm, and the retention factor *k*_propranolol_ = 5.50.

1-(2-naphthyl)ethanol HPLC: the isocratic eluent was 40:60 *V*:*V* acetonitrile: water mixture with a flow rate of 0.6 mL/min. The detection wavelength was 220 nm, and the retention factor *k*_1-(2-naphthyl)ethanol_ = 4.20.

We used standard solutions with a calibration curve or a two-point calibration to determine the binding concentration.
